# Rapamycin golden jubilee and still the miraculous drug: a potent immunosuppressant, antitumor, rejuvenative agent, and potential contributor in COVID-19 treatment

**DOI:** 10.1186/s40643-022-00554-y

**Published:** 2022-06-13

**Authors:** Mohamed A. Mohamed, Waill A. Elkhateeb, Ghoson M. Daba

**Affiliations:** Chemistry of Natural and Microbial Products Department, Pharmaceutical Industries Researches Institute, National Research Centre, El Buhouth St. Dokki, Giza, 12622 Egypt

**Keywords:** Rapamycin, Biosynthesis, Gene cluster

## Abstract

Although celebrating its golden jubilee, rapamycin’s importance keeps increasing by the day. Starting as a promising antifungal agent, then as a potent immunosuppressant, strong anticancer drug, and now rapamycin is attracting serious attention as a rejuvenative agent and a possible contributor in treating this era pandemic, COVID-19. Due to its diverse biological activities and promising medical applications, we aimed in this review to put rapamycin under the spot and highlight its discovery, famous microbial producers, reported biological activities, chemical structure, famous analogues, and biosynthesis. Moreover, discuss some rapamycin production approaches including solid-state fermentation, and stressing out producing strain. On the other hand, describe its action mechanism and trials to use it in treatment of COVID-19. Additionally, we highlighted some of the side effects accompanying its use, and describe some approaches reported to minimize these undesired effects. Finally, we report the current status of rapamycin and its analogues in global market, and discuss future prospects of this potent drug.

## Introduction

**Fig. 1:**
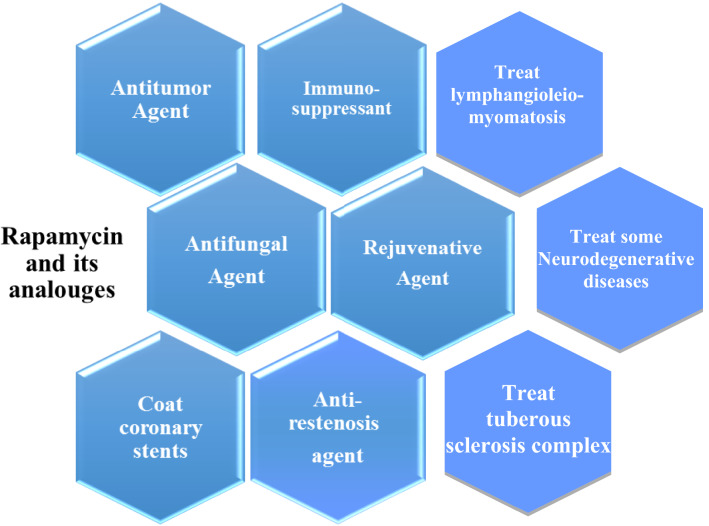
Some uses of rapamycin and its analogues

Immunosuppressants are important chemical compounds that keep immune system under control, especially after organ transplantation to prevent rejection of organ. Also, immunosuppressants are used to treat autoimmunity-related diseases (Fireman et al. 2004). Currently, some immunosuppressants are repurposed to contribute in treatment of COVID-19 and similar diseases that develop cytokine storm (Patocka et al. 2021). Out of the known immunosuppressants, rapamycin (also known as sirolimus) is ranked among the most potent immunosuppressants. Rapamycin exerts also many bioactivities as shown in Fig. 1, such as antitumor, anticancer, antiviral, antifibrotic, anti-inflammatory, antiproliferative, antiangiogenic, lifespan extension, antiaging, neuroregenerative, and neuroprotective activities (Nührenberg et al. 2005; Cantaluppi et al. 2006; Cloughesy et al. 2008; Song et al. 2015; Yoo et al. 2017; Mohamed et al. 2019a; Maiese 2020; Martel et al. 2021). Additionally, it contributes in treating acute myeloid leukemia, retinal and choroidal vascular diseases (Yang et al. 2007; Ekici et al. 2007). Moreover, rapamycin had many approvals by the American Food and Drug Administration (FDA) to be used in treatment of many diseases. So rapamycin was first discovered as antifungal agents, then known for its potent diverse bioactivities including its immunosuppressing effect, anticancer and antitumor potentials. Currently, there is a spreading trend for repurposing rapamycin in treatment of this era disease, COVID-19. Hence, we aimed in this review to highlight the history, chemical structure, different analogues, biosynthesis, production, and action mechanism of rapamycin.

### History of rapamycin discovery, biological activities, and FDA approvals

Rapamycin is a macrolide that was first discovered in 1972 as a product of the actinomycetes strain *Streptomyces hygroscopicus* in the Easter Island (natively known as Rapa Nui island) in Chile (Vézina et al. 1975). It was first known as antifungal agent against *Candida* spp. that showed activity stronger than antifungal activity achieved by amphotericin B in murine model of systemic candidiasis (Baker et al. 1978). Following studies revealed that rapamycin exhibits antitumor effect against mammary, B16 43 melanocarcinoma, colon 26, and EM ependymoblastoma cell lines (Douros and Suffness 1981; Garber 2001). Later, it was observed that this potent polyketide has strong immunosuppressive effect through inhibiting cell proliferation of antigen-induced T cell and B and antibody formation, which made rapamycin used in patients after organ transplantation, then it was licensed in 1999 by FDA as a prophylaxis agent for renal rejection (Seto 2012). Although the discovery of rapamycin was in 1975, the year 1995 is considered as the golden year for this potent polyketide as lots of studies were published focusing on rapamycin production, biosynthesis, and elucidation of its action mechanism (Kojima et al. 1995; Cheng; Fang and Demain, 1995; Nlshida et al. 1995; Dumont and Su 1995). However, rapamycin continues to attract attention and keep updating its uses, as it was repurposed after the spread of the pandemic COVID-19. Also, it got a new FDA approval in 2021 to be used for treatment of some tumors. Actually, apart from FDA approvals recorded for its analogues, rapamycin itself has four FDA approvals in its record, the first was in 1999 for preventing kidney rejection after transplantation, and the second was in 2003 to be used as antirestenosis agent that prevents restenosis of coronary arteries after angioplasty procedures due to its strong inhibiting effect on the proliferation of vascular smooth muscle cells (Tsang et al. 2007; Hambright et al. 2020). Later, rapamycin got also FDA approval for promoting life span in different organisms and delaying many age-related diseases (Arriola Apelo et al. 2016a). The fourth FDA approval was in 2021 for treatment of locally advanced metastatic or unresectable malignant perivascular epithelioid tumors (Świtaj et al. 2021).

### Chemical structure of rapamycin and its analogues (rapalogues)

**Fig. 2:**
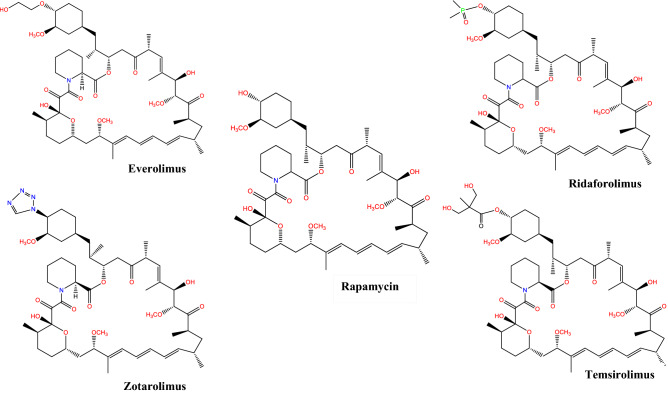
Chemical structure of rapamycin and some of its analouges

The structure of rapamycin was detected using X-ray crystallography together with ^1^H and ^13^C NMR analysis (Paiva et al. 1991). Rapamycin molecule consists of a very large lactone ring (macrolide ring) that contains α-ketonic group. This ring is formed through condensation of propionate and acetate together with the polyketide ring (Fig. 2). The ring backbone carries 3 conjugated double bonds. A sole nitrogen atom exists in this polyketide. Moreover, rapamycin structure carries 6 membered hemiketal ring (C_10_ to C_14_). Out of lactone ring, a tri-substituted cyclohexane ring (C_37_ to C_42_) is found (Paiva et al. 1991). Many analogues were derivatized from rapamycin (Fig. 2). Everolimus is the 2-hydroxyethyl derivative of rapamycin that is used mainly as an immunosuppressant that targets causes of chronic allograft dysfunction or late graft loss (Nashan 2002). It is the first oral inhibitor of mammalian target of rapamycin (mTOR), and its mode of action resemble that of rapamycin, as it blocks interleukin (IL)-2- and IL-15-driven proliferation of B cells, T cells, vascular smooth muscle cell through inhibition of p70 S6 kinase activation, which results in arresting the cell cycle at G1 phase, and preventing cells progression to S phase (Nashan 2002; Chapman and Perry 2004). This inhibitory action is mediated via formation of a complex together with immunophilin FK506-binding protein 12 (FKBP12) in a potency threefold higher than rapamycin does (Schuler et al. 1997; Chapman and Perry 2004). It got FDA approval in 2009 for treating advanced kidney cancer (Atkins et al. 2009).

Zotarolimus (known also as ABT-578) is another derivative of rapamycin (Fig. 2). It is semi-synthetically developed to also prevent restenosis (Burke et al. 2006; Vedantham et al. 2010). Zotarolimus has a shorter in vivo half-life with potency that is comparable to that of rapamycin (Chen et al. 2007). Zotarolimus differs from everolimus by having tetrazole group adjacent to the methoxy group instead of hydroxyethyl substituent existing in everolimus. Ridaforolimus (previously known as deforolimus) is a nonprodrug analogue of rapamycin, where a phosphate group substituted the secondary alcohol moiety at C_43_ (Dancey and Monzon 2011). Ridaforolimus was designed to improve solubility and oral delivery (Mita et al. 2008), and treat bone sarcoma or metastatic soft tissue sarcoma (De Vera and Reznik 2019). Ridaforolimus inhibits cell growth, metabolism, division, and angiogenesis. It has antitumor activity against different in vitro and in vivo models (Rivera et al. 2011). Temsirolimus (also known as Rapamune) is a soluble ester derivative of rapamycin. In 2007, temsirolimus became the first mTOR-targeting agent that obtained FDA approval for treating patients having advanced renal cell carcinoma (Le Tourneau et al. 2008). Unlike everolimus and zotarolimus, temsirolimus is given intravenously due to its poor absorption when orally administered (Boni et al. 2009), and majority of temsirolimus is excreted in feces and around 5% is excreted in urine (Omae et al. 2016).

### Biosynthesis of rapamycin

Rapamycin biosynthesis was described in many studies (Reynolds and Demain 1997; Mohamed et al. 2019a). Concerning precursors involved in rapamycin biosynthesis, the macrolide ring characterizing rapamycin is synthesized from seven propionate units together with seven acetate units, while methionine is the precursor from which the three O-methyl groups are derived (Paiva et al. 1991). On the other hand, the heterocyclic ring existing in rapamycin structure originates from pipecolic acid that is synthesized from lysine, while cyclohexane moiety is derived from the shikimic acid pathway (Paiva et al. 1993a; b). Rapamycin biosynthetic mechanism begins with the cyclohexane ring to which acetate and propionate molecules are added to construct a polyketide backbone in a head-to-tail way. Later, the pipecolate is linked to the polyketide chain, and the ring is closed by lactone formation. The 3 methyl groups are then transferred from methionine by S-adenosyl methionine to make the three methoxy groups (Mohamed et al. 2019a).

**Fig. 3:**
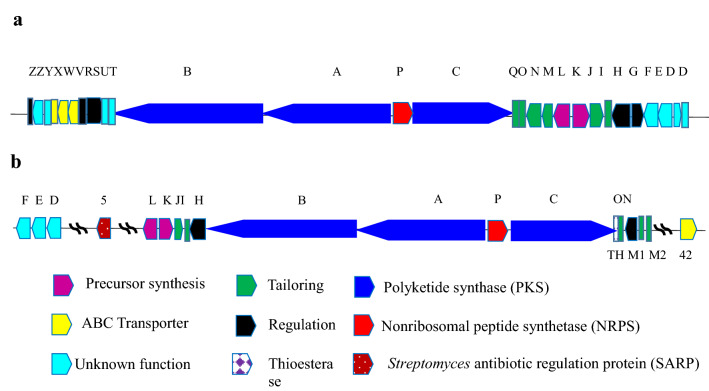
Biosynthetic gene clusters of rapamycin in *Streptomyces hygroscopicus* ATCC29253 (**a**), and *Actinoplanes* sp. N902-109 (**b**)

The gene cluster involved in rapamycin biosynthesis in strain *Streptomyces rapamycinicus* NRRL 5491 was reported and deposited in GeneBank under the accession number X86780 (Schwecke et al. 1995). The rapamycin biosynthetic gene cluster of *Streptomyces hygroscopicus* ATCC29253, and *Actinoplanes sp. N902-109* are shown in Fig. 3, and reported or putative function of rapamycin production genes in *Streptomyces hygroscopicus* ATCC29253 are shown in Table (1). The difference between the rapamycin biosynthetic gene clusters in these two rapamycin producers (*Streptomyces hygroscopicus* ATCC29253 and *Actinoplanes* sp. N902-109) was studied by Huang et al. (2015). In that study, sequence and organization of the genes were compared using different softwares including Glimmer 3.02, anti-SMASH, and Subsystem Technology. Organization of some genes (the multifunctional polyketide synthases (PKS) genes *RapA, RapB, RapC*, and NRPS-like *RapP*). Differences appeared in organization of precursor synthesis genes and macrolactone modification flanked the PKS core region in N902-109, while their homologs in ATCC29253 were located downstream of the PKS core region. Moreover, ATCC29253 gene cluster lacked homolog of gene encoding a putative type II thioesterase responsible for over-production of rapamycin in N902-109. Additionally, ATCC29253 gene cluster lacked also homologs of gene *rapQ* encoding a methyltransferase in N902-109, and *rapM* gene was discovered instead of it in ATCC29253. As shown in Table 1, PKS genes are *rap A, B*, and *C*, while nonribosomal peptide synthetase NRPS-like gene is *rapP*. The precursor synthesis genes responsible for precursor synthesis, macrolactone regulation, and tailoring (*rapO*, *rapN*, *rapM*, *rapL*, *rapK*, *rapJ*, *rapI*, *rapH*, and *rapG*) are located downstream of the PKS genes (Huang et al. 2015). Studying and understanding rapamycin biosynthetic gene cluster can help in constructing many rapamycin bioactive analogs and increase its productivity through conducting genetic manipulation and precursor-directed biosynthesis (Graziani et al. 2003; Gregory et al. 2004; Mohamed et al. 2019a).

**Table 1:** Rapamycin biosynthetic genes in *Streptomyces hygroscopicus* ATCC29253

Gene	Size (aa)	Putative function
*rapA*	8563	Polyketide synthase (PKS)
*rapB*	10223	Polyketide synthase (PKS)
*rapC*	6020	Polyketide synthase (PKS)
*orfD*	387	Unknown
*orfE*	465	Unknown
*orfF*	453	Transporter
*rapG*	330	Regulation
*rapH*	872	Regulation
*rapI*	260	Modification
*rapJ*	386	Modification
*rapK*	334	Starter unit biosynthesis
*rapL*	345	Precursor synthesis
*rapM*	317	Encoding a methyltransferase
*rapN*	404	Modification
*rapO*	77	Modification
*rapP*	1541	Nonribosomal peptide synthetase (NRPS)
*rapQ*	317	Modification

### Rapamycin production

Out of all reported producers of rapamycin, the actinomycetes genus *Streptomyces* is the most famous and predominant producer of this potent polyketide. *Streptomyces hygroscopicus* ATCC 29253 (NRRL 5491; AY B-994; DSM 41530; IMET 43975) was the first reported producer of rapamycin (Vezina et al. 1975). Later, two other strains of *S. hygroscopicus* (strain AY B 1206 and strain C9) were identified as rapamycin producers (Kojima et al. 1995). At the same year, rapamycin was also produced by the strain *S. hygroscopicus* FC904 (Nlshida et al. 1995). *Actinoplanes* sp. N902-109 was also introduced as a producer of rapamycin that brock the rapamycin exclusiveness reported for *S. hygroscopicus* (Nlshida et al. 1995). Production of rapamycin is commonly performed using shaken flasks inoculated with *Streptomyces hygroscopicus* in specific production medium containing mannose and/or fructose as carbon sources (Kojima et al. 1995; Mohamed et al. 2019b). Moreover, addition of amino acid such as L-lysine positively increases production yield (Cheng et al. 1995). On the contrary, amino acids as phenylalanine and methionine had negative influence on rapamycin production (Mohamed et al. 2019b). Few studies have described production of rapamycin in complex media such as medium containing shikimic acid which failed to increase titer of rapamycin (Fang and Demain 1995). Different approach was conducted by Mohamed et al. (2019b), who studied the effect of stressing-out *Streptomyces hygroscopicus* ATCC 29253 on rapamycin production. Using medium supplemented with 1% NaCl caused 56.4% increase in obtained rapamycin yield. Moreover, the yield reached 129.7% by using 1.5-fold concentrated production medium, and higher yield (132%) was achieved when incubation was under fluctuated temperatures. Many other stressing-out factors didn’t affect rapamycin production such as repeated inoculation of producer strain, adding camel milk to medium components, using nano-sized soymeal, and coculturing with sensitive strain such as *Candida albicans* (Mohamed et al. 2019b). Solid-state fermentation (SSF) is fermentation technique that is used in pharmaceuticals, food, textile and other industrial fields. SSF is used instead of liquid medium and is characterized by being simpler, cost-saving, results in higher productivity, shorter time of production, lower energy, and requiring reduced volumetric size (Manan and Webb 2018). On the other hand, SSF requires studying behavior of several parameters related to transfer of mass, gases, and heat through the small solid medium particles (Krishna 2005; Singhania et al. 2009).

Many biologically active compounds in general, and macrolides in particular have been produced using SSF including erythromycin, carbomycin, tetracycline, oxytetracycline, oleandomycin, spiramycin, pimaricin, and tylosin. However, studies describing production of rapamycin using SSF are quite rare. Khedkar et al. (2004) used *S. tsukubaensis* for rapamycin production using SSF medium. In this patent, the medium consisted of wheat rava, wheat bran, oat meal, broken wheat, boiled rice, rice rava, beaten rice, maize bran, maize grits, oat bran, bagasse, tapioca residue, soy grits, soy flakes, rice flakes, ceramic beads, glass beads, sponge or mixture of any of them. Khedkar et al. used carbon source from glucose, different source starches, sucrose, maltose, malto-dextrin, soybean oil, acetate or mixture of any of the above-mentioned carbon sources. On the other hand, they used nitrogen source from ammonium sulphate, dried yeast, yeast extract, casein hydrolyzate, soy peptone, bacteriological peptone, cotton seed flour, corn steep liquor or mixture of any of the above-mentioned nitrogen sources. Chiang (2015) has studied the effect of adding 4 g/L carboxymethylcellulose (CMC) on rapamycin production, which resulted in obtaining 323 mg/L of rapamycin compared to 165 mg/L in the control without CMC. Also, Chiang conducted SSF using 8 substrates in order to explore their effect on rapamycin production. As result, barley caused remarkable increase in rapamycin production (393 mg/kg), while maximum rapamycin yield (525 mg/L) was achieved by using solid barley. On the other hand, Chiang has also studied the static adsorption of rapamycin using macroporous adsorption resin, MAC-3 (0.5 g of resin), which increased rapamycin production to 700 mg/L.

### Rapamycin action mechanism

**Fig. 4:**
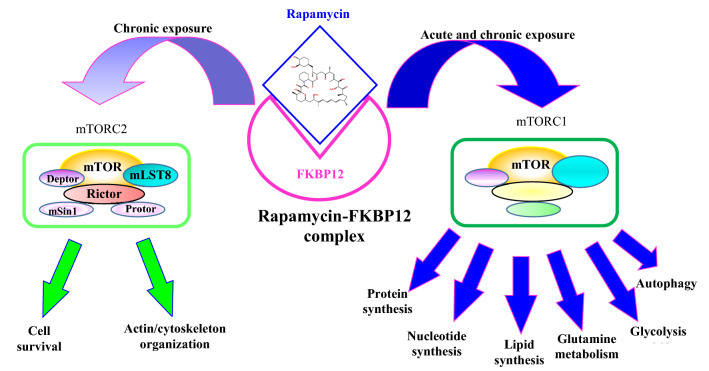
The two functional mTOR complexes and their role in regulating main cellular processes

The molecular receptor (target) of rapamycin is known as mammalian target of rapamycin (mTOR), which is a P13K-related protein kinase that play an important main role in regulation of different cell activities such as protein synthesis, cell cycle progression, cell survival and proliferation (Savvoulidis et al. 2019). This regulatory effect is based on inhibiting multiple downstream actions of mTOR’s activity including synthesizing components (as ribosomes) needed for synthesis of macromolecular, increasing cell size, and progression of cell cycle through the G1 phase (Savvoulidis et al. 2019). Generally, mTOR has two functionally distinct complexes (mTORC1 and mTORC2) as illustrated in Fig. 4. The first one (mTORC1) integrates different signals from growth factors, energy levels, nutrients, and oxygen such as amino acids to promote cell growth and proliferation by activation of various anabolic processes such as lipid synthesis, protein synthesis, and nucleotide synthesis. Moreover, stimulates energy metabolism processes such as glutamine metabolism and glycolysis. Also, inhibits some catabolic processes as autophagy. On the other hand, the second complex (mTORC2) only responds to growth factors and regulates both cell survival and actin/cytoskeleton organization. It

 should be noted that rapamycin acutely inhibits mTORC1, while chronic exposure to rapamycin can results also in inhibiting mTORC2 (Fig. 4). The action mechanism of rapamycin allows it to interrupt cytokine signaling which stimulates growth and differentiation of lymphocytes. This process begins when rapamycin binds to, and forms a complex with a cytosolic specific protein known as FK506 binding protein 12 (FKBP12). The resulting complex interacts with mTOR and blocks progression of the cell cycle of T cells that causes suppression of T-cell proliferation that is induced by cross-linking of antigenic peptides, T-cell receptors, or cytokines as interleukins (Dumont and Su 1995; Park et al. 2010). Dumont and Su (1995) have described two main biochemical changes that take place during this mode of action. As a start, rapamycin binds to FKP12, and form a complex that physically interacts with mTOR which is acting upstream of two enzymes. The first one is enzyme p70 S6 kinase (p70s6k) which is inhibited causing an early response of cytokine-induced mitogenesis. After inhibiting p70s6k (its main substrate is 40S ribosomal subunit S6 protein), rapamycin starts to decrease translation of some mRNA encoding for elongation factors and ribosomal proteins and consequently decreases protein synthesis. On the other hand, the second enzyme is cyclin-dependent kinase cdk2-cyclin E complex, which is responsible for regulating Gl/S transition. This enzyme inactivation is caused by preventing decline of its inhibitor (p27 cdk) which normally follows stimulation of IL-2.

### Repurposing rapamycin for COVID-19

The spread of COVID-19 pandemic caused by human coronavirus-2, and its related high mortalities have left the world helpless without any effective cure. As there is no time to waste on screening for novel compounds as a sole solution of this disease, current trends are focusing on repurposing and testing available drugs (known for treating other diseases) and check their activity against corona virus. The idea of testing the anti-COVID-19 activity of rapamycin came from its ability to inhibit synthesis of protein, and expression of pro-inflammatory cytokines (as IL-2, IL-6 and, IL-10). Hence rapamycin can, from theoretical point of view, inhibit viral synthesis, prevent cytokine storms. Therefore, rapamycin was evaluated for its anti COVID-19 activity (Husain and Byrareddy 2020), and some studies have described its potency as promising candidate for treating COVID-19 when compared with available antiviral medications because its efficiency may not be affected by high mutation rate of viral RNA, which made antiviral drugs helpless.

Some studies have reported the use of rapamycin and other mTOR for calming down cytokine storms and preventing progression of COVID-19 (Omarjee et al. 2020; Patocka et al. 2021). Other studies have described the effect of combining the action of rapamycin together with different drugs such as metformin, lithium chloride, or calcineurin inhibitors (Khalil 2020; Hasbal et al. 2021; Karp 2021). The rejuvenative potentials of rapamycin and rapalogues (Guarda et al. 2004; Blagosklonny, 2007; Lamming et al. 2013; Dai et al. 2014; Ghasemnejad-Berenji 2021) were also suggested to be another reason to use them in reducing severity and impacts of infection with COVID-19 (Husain and Byrareddy 2020; Guarda et al. 2014; Blagosklonny 2020; Bischof et al. 2021). However, further studies are critically needed to evaluate and consider consequences of using mTOR drugs as rapamycin against COVID-19.

### Some adverse reactions accompanying treatment with rapamycin and rapalogues

Although rapamycin and rapalogues are used in treatment of different diseases and in decreasing chances of organ rejection after organ transplant, many side effects were reported accompanying their use. Generally, adverse reactions represent a major concern that sometimes can limit application of any drug. Side effects reported for using rapamycin in patients having organ transplant to avoid organ rejection includes thrombocytopenia, immunosuppression, impaired wound healing, stomatitis, glucose intolerance, high serum and cholesterol triglycerides (Kaeberlein 2013; Johnson and Kaeberlein 2016; Arriola Apelo et al. 2016b). Being an immunosuppressant is commonly accompanied with an elevated risk of infection or even cancer due to the ability of rapamycin and rapalogues to suppress some tumor immune mechanism (Dumas and Lamming 2020). It should be noted that chances of occurrence of such side effects increases when rapalogues are given at a high dose and chronically. However, applying some techniques during treatment with rapamycin can minimize side effects. Such techniques focus on using local rapamycin treatment instead of its systemic administration, or applying an intermittent rapamycin dosing schedule, or using rapalogues instead of rapamycin itself. Falke et al. (2015) have reported that administrating microspheres loaded with rapamycin under the kidney capsule of ureter-obstructed rats inhibited local fibrotic response and decreased systemic adverse effects of rapamycin.

On the other hand, Arriola Apelo et al. (2016a) have mentioned that some rapamycin side effects are mediated by the inhibition of a second mTOR-containing complex that is less sensitive to rapamycin (other than the known complex formed when rapamycin interact with FKP12). Hence, it was suggested that compounds targeting the first complex in a more specific way may reduce majority of mediated side effects. Arriola Apelo et al. have also identified an intermittent rapamycin dosing schedule with a reduced adverse effects when compared to daily treatment with rapamycin especially on immune system, glucose and pyruvate tolerance, insulin levels, and fasting glucose. Similarly, many studies have described the safe toxicity profile of rapalogues with reduced side effects on glucose and pyruvate tolerance (Pallet and Legendre 2013; Arriola Apelo et al. 2016a; Su et al. 2016). However, some side effects indeed reported after treatment with rapalogues such as mucositis, skin rashes, anemia, fatigue, neutropenia, and metabolic disorders such as hypercholesterolemia, hypertriglyceridemia, and hyperglycemia, pulmonary toxicity, secondary lymphoma (De Masson et al. 2011; Paplomata et al. 2013; Cheaib et al. 2015; Guimarães et al. 2015; Viana et al. 2018; Arena et al. 2021; Dang et al. 2021).

### Rapamycin in drug market

**Fig. 5:**
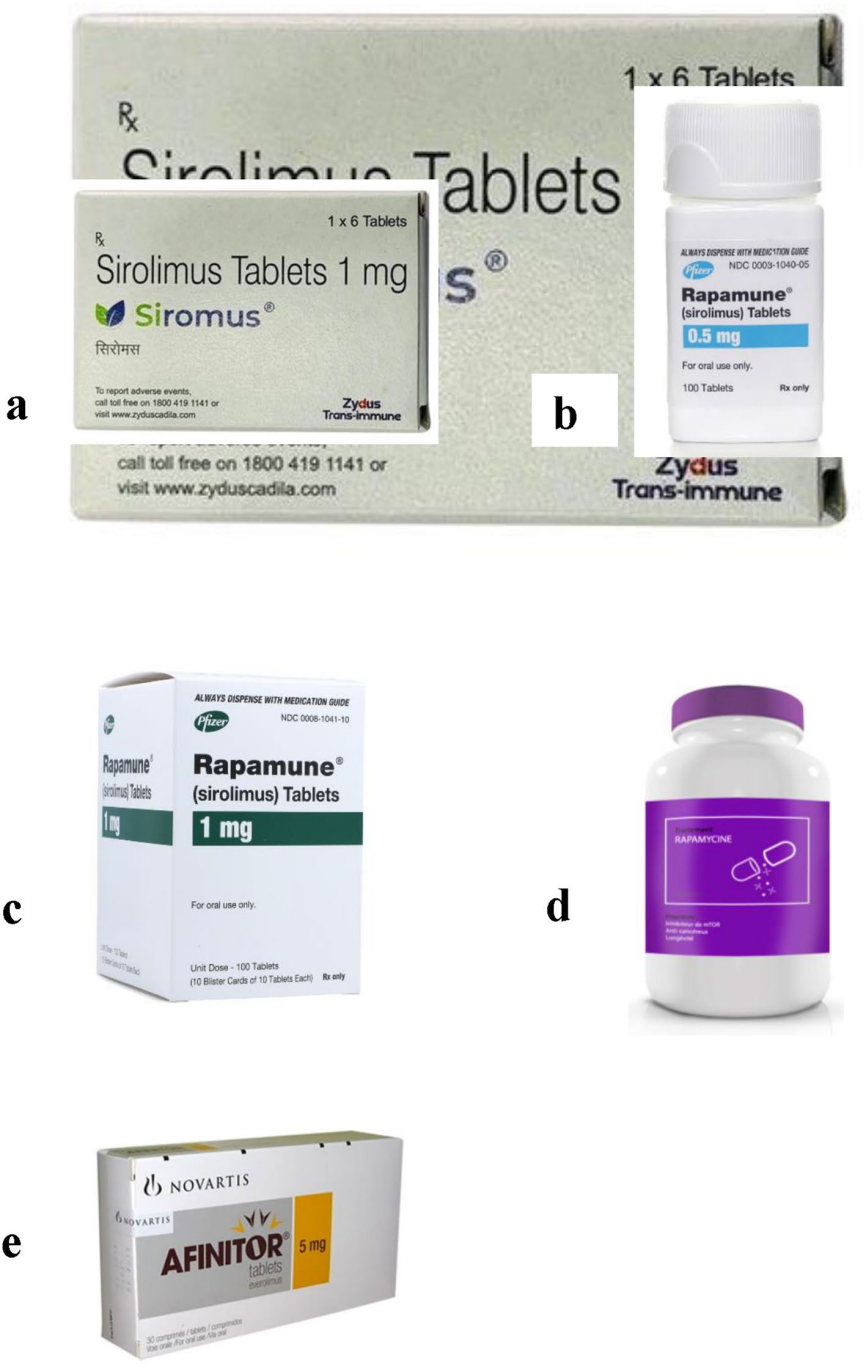
Some rapamycin products. Sirolimus tablets 1 mg https://www.indiamart.com/ (**a**), Rapamune 0.5 mg https://www.indiamart.com/ (**b**), Sirolimus 1 mg https://www.wiitus.com/ (**c**), Rapamycine https://www.longlonglife.org/ (**d**), and Afinitor (5 mg) (**e**)

The insufficient availability of rapamycin is the main reason for its high price. The global market of rapamycin was USD 275.93 Million in 2020 and is expected to reach USD 302.77 Million by the year 2028. The price of oral tablets of sirolimus (concentration of 0.5 mg) is about $226/30 tablets, which is expensive when compared with other generic drugs. Rapamycin is produced commercially under many trade names such as Rapamune^®™^ is released in markets in 1999 and come in two different formulations: oral solution, and coated tablets. Sirolimus^®^ 1 mg, Rapamune 0.5 mg, Rapamune 1 mg and Rapamycine^®^. Other derivatives of rapamycin such as everolimus; temsirolimus; and zotarolimus under the trade names Certican^®^, Afinitor^®^, Xience V^®^, Endeavor^®^, and Torisel^®^ are also available (Fig. 5).

## Conclusion and future prospects

Although it is discovered in the seventies of the previous century, rapamycin is still considered as miraculous drug that started as antifungal agent, then its diverse biological activities and therapeutic potentials were explored. Understanding potency of this drug has nominated it for repurposing as a potential treatment or support to other COVID-19 treatment. Extensive investigations are required to evaluate impact of such approach. Constructing a database website that carry all updated studies and trials conducted in this field is extremely important, as it will decrease chances of repeating approaches that were tried by other scientific groups, and reduce the time consumed by researchers moving between different search engines and scientific websites to check for scientific data available for a certain topic.

On the other hand, further studies are needed to increase productivity of this important drug. Solid-state fermentation, although still not commonly applied, represents promising approach for increasing production of rapamycin. Similarly, stressing out producer strain, genetic manipulation can have important roles in achieving such a purpose.

## Data Availability

Not applicable.
